# Evidence on the Effectiveness of Water, Sanitation, and Hygiene (WASH) Interventions on Health Outcomes in Humanitarian Crises: A Systematic Review

**DOI:** 10.1371/journal.pone.0124688

**Published:** 2015-09-23

**Authors:** Anita Ramesh, Karl Blanchet, Jeroen H. J. Ensink, Bayard Roberts

**Affiliations:** 1 Department of Clinical Research, Faculty of Infectious Tropical Diseases, London School of Hygiene & Tropical Medicine, London, United Kingdom; 2 Department of Disease Control, Faculty of Infectious Tropical Diseases, London School of Hygiene & Tropical Medicine, London, United Kingdom; 3 Department of Health Services Research and Policy, Faculty of Public Health and Policy, London School of Hygiene & Tropical Medicine, London, United Kingdom; Catalan Institute for Water Research (ICRA), SPAIN

## Abstract

**Background:**

Water, sanitation, and hygiene (WASH) interventions are amongst the most crucial in humanitarian crises, although the impact of the different WASH interventions on health outcomes remains unclear.

**Aim:**

To examine the quantity and quality of evidence on WASH interventions on health outcomes in humanitarian crises, as well as evaluate current evidence on their effectiveness against health outcomes in these contexts.

**Methods:**

A systematic literature review was conducted of primary and grey quantitative literature on WASH interventions measured against health outcomes in humanitarian crises occurring from 1980–2014. Populations of interest were those in resident in humanitarian settings, with a focus on acute crisis and early recovery stages of humanitarian crises in low and middle-income countries. Interventions of interest were WASH-related, while outcomes of interest were health-related. Study quality was assessed via STROBE/CONSORT criteria. Results were analyzed descriptively, and PRISMA reporting was followed.

**Results:**

Of 3963 studies initially retrieved, only 6 published studies measured a statistically significant change in health outcome as a result of a WASH intervention. All 6 studies employed point-of-use (POU) water quality interventions, with 50% using safe water storage (SWS) and 35% using household water treatment (HWT). All 6 studies used self-reported diarrhea outcomes, 2 studies also reported laboratory confirmed outcomes, and 2 studies reported health treatment outcomes (e.g. clinical admissions). 1 study measured WASH intervention success in relation to both health and water quality outcomes; 1 study recorded uptake (use of soap) as well as health outcomes. 2 studies were unblinded randomized-controlled trials, while 4 were uncontrolled longitudinal studies. 2 studies were graded as providing high quality evidence; 3 studies provided moderate and 1 study low quality evidence.

**Conclusion:**

The current evidence base on the impact of WASH interventions on health outcomes in humanitarian crises is extremely limited, and numerous methodological limitations limit the ability to determine associative, let alone causal, relationships.

## Introduction

Diarrheal disease—nearly 90% of which has been attributed to suboptimal water, hygiene, and sanitation (WASH)—is one of the largest causes of morbidity and mortality in children under five years of age in low and middle-income countries, where it kills more children than HIV, malaria, and measles combined.[1] WASH interventions aim to prevent and control transmission routes of bacteria (e.g., *Shigella*, *E*. *coli*) viruses (e.g., cholera, hepatitis A and E) and parasites (e.g., *Cryptosporidium*, soil transmitted helminths) to new human hosts.[2–6] Evidence from non-emergency settings demonstrates that poor and unsafe access to water, sanitation, and hygiene (WASH) plays a key role in the transmission of diarrheal disease.[2, 5, 7] There has been a continuing dialogue on the relative ability of different WASH interventions (e.g., safe water storage for potable water, latrines for sanitation, and soap for hygiene/hand washing) to reduce diarrhea.[8] While it is generally acknowledged that hand washing with soap promotion may reduce diarrhea by up to 40% in non-emergency settings, the impact of WASH interventions on diarrhea is disputed, with estimates of diarrheal reductions ranging from 15–50% depending on reporting and publication bias.[5, 8, 9]

In humanitarian crises, WASH are amongst the principal challenges—particularly in the acute and early recovery phases, when diarrheal disease has been found to account for nearly 40% of deaths in camp residents and 80% of deaths in children under two years of age.[10–12] A recent review of infectious disease outbreaks after natural disasters highlighted the role of WASH in relation to a majority of disease outbreaks.[13] Water related pathogens (cholera, *Shigella*) were responsible for 85% of the 50,000 deaths after the sudden influx of 800,000 refugees from Rwanda into the Democratic Republic of Congo in July 1994.[11, 14] More recent large-scale outbreaks of cholera (e.g. Haiti, 2010) and hepatitis E (e.g., South Sudan, 2011) have demonstrated the absolute necessity of rapid and efficient deployment of WASH interventions in complex emergency settings.[15–17]

WASH professionals operating in humanitarian response must be able to deliver interventions ranging from safe and sufficient drinking water provision to efficient wastewater and excreta removal methods in extremely unstable and insecure contexts.[18] Complex emergencies differ from stable settings in a variety of ways, ranging from population dynamics to the actual types of interventions that are possible to deploy (e.g., installing complex sanitation structures in temporary sites with shifting water tables). As a result, it is important to assess how well WASH interventions perform in humanitarian settings. While a few studies have evaluated WASH interventions (e.g., water filters) in these contexts, the majority of WASH intervention research appears to have measured intervention success against water quality outcomes.[19–25] Thus, it has been unclear how much of the evidence base on WASH intervention in humanitarian crises relates directly to health outcomes (e.g., diarrhea).

The overall aim of this systematic literature review was to examine the quantity and quality of evidence on WASH interventions on health outcomes in humanitarian crises, as well as evaluate what the current evidence indicates about the effectiveness of WASH interventions on health outcomes in these contexts.

## Methods

The study followed standard systematic review methodology, and adheres to the Preferred Reporting Items for Systematic Reviews and Meta-Analyses (PRISMA) statement.[26] Two independent, blinded readers conducted the review from search to paper selection and quality grading; a third reader was consulted in event of disagreement at any stage of the review.

### Inclusion and exclusion criteria

Studies were selected or excluded for inclusion based on the criteria listed in Table 1.

**Table 1:** Inclusion and exclusion criteria.

Category	Included	Excluded
Intervention type	WASH related intervention intended to improve health outcomes (usually diarrheal disease).	Studies with no specific health intervention (i.e., examining only health needs, prevalence, health risk-factors, and co-ordination)
Populations of interest	Populations affected by humanitarian crises and receiving humanitarian assistance (including refugees and internally displaced persons), in low and middle-income countries (based upon World Bank country classification, 2012).^a^	Studies that examined preparedness and resilience not linked to health outcomes in humanitarian crises (e.g. studies on sanitation fortification before flooding)
Phase of humanitarian crises	Studies that occurred during humanitarian crises, e.g., measuring: i) the impact of preparedness and resilience on public health outcomes during a humanitarian crises and/or ii) studies that evaluate the impact of public health interventions during the acute, chronic, or early recovery phases of humanitarian crises.^b^	Studies that occurred pre or post conflict of a humanitarian crisis (e.g. preparedness, resilience) that do not measure the outcome or intervention of interest during the actual humanitarian event.
Study types and designs	Quantitative studies including: randomized and non-randomized controlled trials as well as controlled before-after, interrupted time series, and economic studies (cost-effectiveness, cost-utility, cost-benefit, economic modelling)	Qualitative studies (e.g. on process and perception of interventions); quantitative studies not measuring a change in health outcomes
Health outcomes and outputs of interest	Primary outcomes (e.g. morbidity, mortality, disease status), secondary outcomes (e.g. soap uptake rate), and primary outputs (e.g. chlorine tablets provided etc.)	
Publication dates	January 1, 1980—April 30, 2013.	
Publication language(s)	English, French.	

^a^ World Bank (2012). Country and Lending Groups, Low and Lower Middle Income countries [cited August 29, 2013]; Available from: http://data.worldbank.org/about/country-classifications/country-and-lending-groups.

^b^ World Health Organization (WHO). Humanitarian Health Action Dictionary. 2013 [cited August 29, 2013]; available from: http://www.who.int/hac/about/definitions/en/index.html.

### Data sources, search terms, and paper selection

Peer reviewed literature was searched via the electronic databases of Embase, Global Health, and Medline via the full list of terms provided in S1 Appendix. Grey literature was searched using similar terms where possible; these additional electronic sources are listed in S2 Appendix. The above were supplemented by reviewing the reference lists of articles selected (‘references of references’) in order to find any other relevant papers. Finally, experts in the field of WASH and humanitarian crises were consulted regarding literature that may have been missing from the search results.

The search structure itself incorporated terms related to (i) terms related to humanitarian crises/early recovery; AND (ii) terms related to public health interventions; AND (iii) terms related to lower and middle income economies; AND (iv) terms related to water, sanitation, and hygiene (WASH).

The systematic literature was conducted in five stages as follows:

*Stage I*: electronic database search; results imported into reference management software; duplicates removed.
*Stage II*: title and abstract review (2a); manuscript review (2b); studies removed via exclusion criteria (Table 1); paper selection; reference review of papers selected
*Stage III*: grey literature review; studies removed via exclusion criteria; paper selection; reference review of papers selected
*Stage IV*: final paper selection, data extraction, and quality assessment. For quality assurance, a second peer reviewer corroborated study selection and data extraction from Stages III and IV.

### Data extraction, analysis, and quality assessment

Once selected, the following data was extracted from each paper into an Excel database: (i) study authors or agency, year; (ii) study country; (iii) setting: urban or rural; (iv) population type (refugee; internally displaced; entrapped population; host population); (v) humanitarian crises type (armed conflict or natural disaster): (vi) health outcome(s) addressed by the public health intervention; (vii) type(s) of public health intervention; (viii) study design; (ix) measurement outcomes (e.g. prevalence, odds ratios etc.); (x) target age group: i) infants: under 6 months; ii) infants and young children: under two years; iii) children under five: 6 months—59 months; iv) school age children: 6 years—15 years; v) adolescents: 10 years—19 years; vi) adults: 20 years—49 years; vii) elderly: 50+ years.

Results were analyzed descriptively as a meta-analysis was not possible due the low number of studies identified and the heterogeneity of interventions and outcome measures. The quality of the final selected studies was evaluated using STROBE guidelines for observational studies and CONSORT guidelines for clinical trials (Table 2) which are widely instruments for assessing study quality.[27, 28] Each instrument is a standardized, itemized checklist of 20–30 items (e.g., details on sample size, discussion of limitations and generalizability, etc.) that are considered to be representative of high quality scientific publication. Each item in these checklists is given equal weight, with some sections (e.g., Methods) containing more checklist items than others; for reference the CONSORT and STROBE checklists are provided, with paper grading, in S3 Appendix and S4 Appendix. Study authors did not use the quality assessment tools to screen out studies as, given the very limited number of studies, it was felt it would be more useful to provide analysis and insight on the quality of all the final selected studies. After independent quality review by two blinded readers, an overall quality score was given to each paper per the STROBE and CONSORT checklists (S3 Appendix and S4 Appendix).[27, 28]

**Table 2:** Published Studies That Measured Public Health Outcomes in Relation to WASH Interventions in Humanitarian Crisis Settings.

Publication [Author, Country]	Population and Crisis	WASH Intervention	Health Outcome	Methods	Results and Quality Rating	Conclusion
Doocy et al (2006), Liberia	**Population:** ID	**Point of use:** Safe water storage; flocculant disinfectant (FD); 200 houses received FD.	**Health outcome:** Diarrhea (non-specific)	**Design:** RCT (unblinded).	**Diarrhea incidence:** 2.8% of weeks in FD houses vs 28.7% of weeks in control houses (P < 0.001). **Diarrhea prevalence:** 38.7% of weeks in control vs 3.5% of weeks in FD houses (P < 0.001). FD houses averaged 0.3 incident weeks and 0.4 prevalent weeks vs 3.2 incident and 4.7 prevalent weeks in control houses (P < 0.001 for both comparisons) over 12 week monitoring period. **Adjusted risk ratios for diarrhea incidence and prevalence:** control vs FD households, incidence: 3.0 (2.7–3.3) and prevalence: 4.4 (4.0–4.8).	*Diarrheal incidence decreased after FD*. *Diarrheal prevalence decreased after FD*. *Flocculant disinfectant appears to be a successful POU intervention in these settings*.
	**Crisis type:** Armed conflict	**Measurements:** *Distribution*: Yes; *Uptake*: No; *Behavior change*: No; *Impact*: Yes	**Definition:** 3 or more loose stools in 24hr period	**Sampling:** 2 arms, 3 blocks each arm (3/7 blocks Camp II; all blocks Camp II). Sample size of 400 households (200 per group) selected to detect 15% difference in diarrhea rate (intervention v control) with statistical assumptions: power = 80%, CI = 95% (alpha = 0.05) and 10% potential loss to follow-up.	**Quality**: High	
	**Crisis stage:** Acute		**How assessed:** *Self-reported* diarrhea via weekly surveys over 12 weeks	**Population enrolled:** 2215 individuals in 400 households (from 2 camps of a total of 22,800 residents); only households with children under 5 were eligible.		
				**Statistics:** Change in incidence, prevalence, adjusted risk ratio; 95% CI; p-values		
Elsanousi et al (2009), Sudan	**Population:** ID	**Point of use:** Household iodinated water filter (IWF); each participant received IWF.	**Health outcome:** Diarrhea (non-specific)	**Design:** Uncontrolled longitudinal study.	**Diarrheal prevalence:** Pre-IWF survey prevalence: 15%; post-IWF survey prevalence: 2.3%. **Diarrheal incidence:** four months prior to IWF: 58 people presented in two weeks; four months post IWF: 6 people presented in two weeks. **Decline in clinic attendance post IWF compared to regional hospitals:** uncorrected X^2:^ 30.71 p<0.0001).	*Diarrheal prevalence decreased after IWF*. *Diarrheal incidence decreased after IWF*. *Iodinated water filtration appears to be a successful POU intervention in this setting*.
	**Crisis type:** Armed conflict	**Measurements:** *Distribution*: Yes; *Uptake*: No; *Behavior change*: No; *Impact*: Yes	**Definition:** 3 or more loose stools in 24hr period	**Sampling:** Convenience sample; all eligible residents given IWF.	**Additional indicators:** Of 647 eligible adult patients, 27 stool samples were submitted: 7 (+) *Giardia lamblia* cysts (giardiasis), 2 (+) *Entamoeba hisolytica* cysts, 4 (+) *Schistosoma mansoni* ova (schistosomiasis), and 2(+) *Taenia saginata* ova (pinworm).	
	**Crisis stage:** Acute		**How assessed:** *Self-report* (primary); *laboratory testing* (limited); *clinic diarrhea admissions* (variable) over 4 mo. study period and 4 mo post IWF	**Population enrolled:** 647 of 713 camp residents (66 residents were aged <2 years) enrolled; 603 remained at study conclusion; clinic admissions for diarrhea 4 months before and after IWF provision.	**Compliance:** Final survey post IWF of 531 participants indicated 86% always used the IWF, 10% occasionally used it, and 4% never used it.	
				**Statistics:** Change in incidence (adjusted), cumulative incidence (attack rate); correlation (uncorrected chi square); 95% CI; p value.	**Quality**: Moderate	
				**Stratification:** Age		
Moll et al (2007), El Salvador, Guatemala, Honduras, Nicaragua	**Population:** IDP	**Point of use:** Safe water storage (provision, upgrades); Latrines (pour, flush, VIP, or composting) WASH education; participants received different interventions and to different degrees, depending on location.	**Health outcome:** Diarrhea (non-specific).	**Design:** Uncontrolled longitudinal study.	**Change in diarrheal prevalence in children <3 years of age:** in 2000, diarrheal prevalences ranged from 25–48%; by 2002, diarrheal prevalences ranged from 11–44%. Six of eight (75%) communities met or greatly exceeded their 2002 goal of reducing diarrheal disease in children <3. **Association between select WASH indicators and diarrhea in children<3 years (univariate):** improved water access [OR = 0.61; 95% CI = 0.47, 0.78; (p<0.0001)]; improved sanitation access [OR = 0.73; 95% CI = 0.57, 0.94; (p = 0.015)]; food preparer hand washing [OR = 0.68; 95% CI = 0.53, 0.90; (p = 0.006)]; child carer hand washing [OR = 0.67; 95% CI = 0.52, 0.87; (p = 0.002)]; *E coli* in stored water [OR = 0.32; 95% CI = 0.11, 0.93; (p = 0.03)]; stored household water covered [OR = 0.58; 95% CI = 0.43, 0.78; (p = 0.0004)]; hand soap available [OR = 0.70; 95% CI = 0.52, 0.94; (p = 0.02)]. The following indicators were found to be associated with diarrheal disease: increasing number of latrine users [OR = 1.08; 95% CI = 1.01, 1.15; (p = 0.03)]; animals having access to water/pumps [OR = 1.48; 95% CI = 1.15, 1.90; (p = 0.002)]. None of these interventions were found to be independently associated with lower diarrheal prevalence.	*Diarrheal prevalences decreased post-intervention rollout*. *Safe water storage*, *improved water access*, *and improved sanitation measures appear protective for childhood diarrhea*. *There is insufficient data from each study to determine if any intervention in this study was independently successful in reducing childhood diarrhea*.
	**Crisis type:** Natural disaster (hurricane)	**Measurements:** *Distribution*: Yes; *Uptake*: No; *Behavior change*: No; *Impact*: Yes	**Definition:** Not provided.	**Sampling:** Evaluation conducted in 2 areas of each of the 4 countries (n = 8 study areas); sample size calculated to detect 25% decrease in diarrhea in children <3 years after WASH interventions (assuming diarrheal prevalence = 25% pre-intervention); sample size calculated with statistical assumptions of power = 80% and CI = 95% (alpha = 0.05) estimated 717 households (800 to account for refusals). Sample size deemed too large, so pooled calculation based on hand-washing (needing largest sample size of all WASH indicators) was used, giving 91 households; to account for refusals, 100 households were enrolled from each site.	**Quality**: High	
	**Crisis stage:** Acute / early recovery		**How assessed:** *Self report*	**Population enrolled:** 800 households (100 from each site that were then pooled for the global diarrhea indicator).		
				**Statistics:** Change in prevalence; 95% CI; p value.		
				**Stratification:** None		
Peterson et al (1998), Malawi (Mozambique refugees)	**Population:** Refugee	**Point of use:** Soap distribution; each participant received soap.	**Health outcome:** Diarrhea (non-specific)	**Design:** Uncontrolled longitudinal study.	**Diarrhea incidence:** Houses with soap on visit days had 27% reduced risk of houses without soap (RR = 0.73, 95% Cl: 0.54–0.98). Houses that used soap on the previous interview day (4 days earlier) had 25% reduced risk of diarrhea than houses without soap (RR = 0.75, 95% CI: 0.51–1.1).	*Diarrheal risk decreased in households when soap was used*. *This study demonstrates that soap provision can significantly reduce diarrheal disease incidence*.
	**Crisis type:** Armed conflict	**Measurements:** *Distribution*: Yes; *Uptake*: Yes; *Behavior change*: Yes; *Impact*: Yes	**Definition:** *new diarrhea*—3 watery stools in 24h by female HoH with no family member having diarrhea in previous 48 hrs; *soap presence*—soap in any form on the day of interview	**Sampling:** Every fourth house eligible, excluded if not home after 2 visits.	**Compliance:** of 402 households, 356 (87%) participated in second survey and 322 (80%) participated in final survey.	
	**Crisis stage:** Early recovery		**How assessed:** *Self report* of diarrhea (2 visits/week over 4 month study period)	**Population enrolled:** 402 houses (represented by 402 female head of households, HoH); 356 households remained enrolled over entire study period.	**Quality**: Moderate	
				**Statistics:** Change in incidence, Mantel Haenszel relative risk and chi square; 95% CIs		
				**Stratification:** Household		
Roberts et al (2001), Malawi	**Population:** Refugee	**Point of use:** Safe water storage (bucket provision); WASH education; 310 houses received buckets.	**Health outcome:** Diarrhea (non-specific)	**Design:** RCT (unblinded).	**Diarrheal Risk:** The 310 houses receiving buckets had 60 diarrheal episodes (AR = 44.5/1000/month) vs 207 diarrheal episodes in 850 control houses (AR = 48.6/1000/week); i.e., 8.4% less diarrhea (not statistically significant). The 51 children <5 years in houses with buckets had 18 diarrheal episodes (AR = 84.3/1000/month) vs. 82 episodes in the 157 children in control houses (AR = 122.4/1000/month); buckets were associated with a 31.1% diarrheal reduction (P = 0.06).	*Diarrheal incidence decreased in households receiving buckets*. *This study suggests safe water storage can reduce diarrheal incidence but the results are borderline significant*. *More research is needed on this type of intervention*.
	**Crisis type:** Armed conflict	**Measurements:** *Distribution*: Yes; *Uptake*: No; *Behavior change*: No; *Impact*: Yes	**Definition:** 3 or more loose stools in 24hr period	**Sampling:** Simple random sampling of every fourth hut in village.	**Diarrheal Association:** Poisson regression indicated buckets in the household (RR = 0.85; p = 0.021) and latrines (RR = 0.87; p = 0.051) were associated with less diarrhea among all age groups. Among children <5, having buckets in the house (RR = 0.57, p = 0.040) was protective against diarrhea.	
	**Crisis stage:** Early recovery		**How assessed:** *Self report* over 4 month study period	**Population enrolled:** 310 individuals selected for intervention; 850 controls enrolled throughout end of study.	**Quality**: Moderate	
				**Statistics:** Cumulative incidence (attack rate, relative risk); p value		
				**Stratification:** Age		
Walden et al (2005), Sudan	**Population:** IDP	**Point of use:** Mass (water) container disinfection (MCD) via chlorination	**Health outcome:** Diarrhea and bloody diarrhea (suspected *Shigella*)	**Design:** Uncontrolled longitudinal study.	**Results:** Outbreak of suspected *Shigella* began early May, MCD occurred last week of June (13,224 containers, an estimated 88% of total, disinfected); watery and bloody diarrhea cases decreased within 5 days post MCD. Cases watery/bloody per week: May Wk 1: 200–210 watery / 90–100 bloody; June Wk 1: 500–510 watery / 210–220 bloody; July Wk 1: 180–190 watery / 180–190 bloody; July Wk 4: 80–90 watery / 0–10 bloody. Statistical associations were not provided.	*Decreased diarrheal incidence corresponded to MCD*. *However*, *it is impossible to establish a causal relationship as statistical associations were not provided*.
	**Crisis type:** Armed conflict	**Measure-ments:** *Distribution*: Yes; *Uptake*: No; *Behavior change*: No; *Impact*: Yes	**Definition:** Not provided.	**Sampling:** Entire camp was selected for MCD (i.e., no sampling).	**Quality**: Low	
	**Crisis stage:** Acute		**How assessed:** *Clinical cases* of watery / bloody diarrhea	**Population enrolled:** 7000 households estimated in camp; each estimated to have ≥2 containers. All houses enrolled for MCD.		
				**Statistics:** Incidence		
				**Stratification:** None		

The PRISMA Checklist for this review, including information on the methodological details above, is provided in S5 Appendix.

## Results

**Fig 1:**
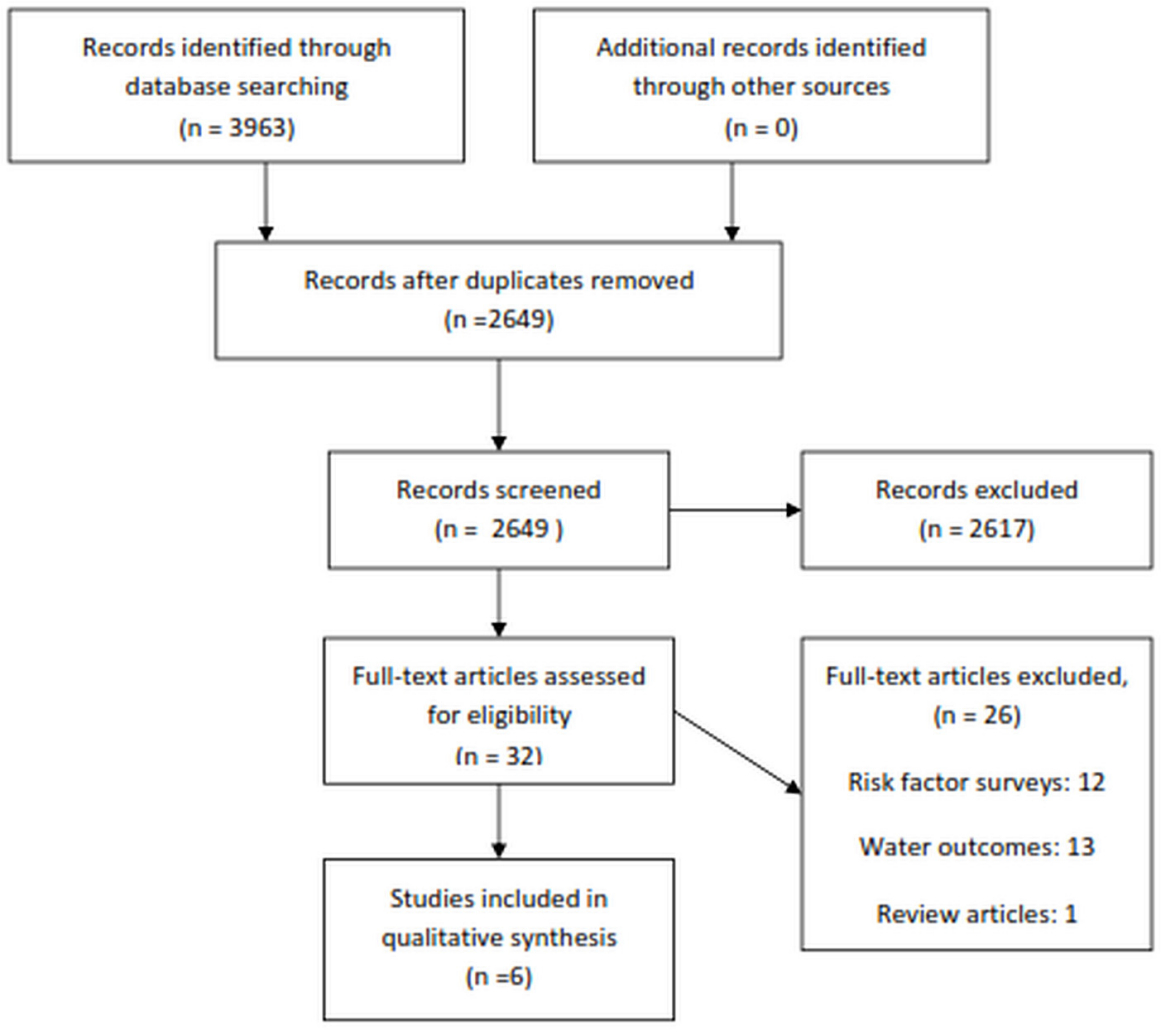
Results of Paper Screening.

The systematic literature review retrieved 3963 articles. After 1314 duplicates were removed, the vast majority of studies (2643 papers) did not occur in humanitarian crises, consider the impact of WASH interventions (e.g., risk factor analysis), or provide measurements related to both interventions and health outcomes. Studies that did not measure health-related outcomes (e.g., diarrhea) but reported the impact of WASH interventions on water quality/purity (e.g., fecal coliform or residual chlorine levels) outcomes were excluded. A total of six published articles met the inclusion criteria (Fig 1).[29–34] Expert consultation yielded no additional studies.

All of the six papers that met the inclusion criteria of this review were conducted within the past 15 years (post 1998 or later), with five [29–31, 33, 34] of the papers published since 2000. No studies identified in the grey literature met the inclusion criteria.

Five [29, 30, 32–34] of the six studies were in Sub-Saharan Africa, and one [31] occurred in Latin America. Five studies occurred in humanitarian crises related to armed conflicts, [29, 30, 32–34] while one occurred in a natural disaster setting in Latin America.[31] Of the five studies in conflict zones, three [29, 30, 34] were conducted amongst internally displaced persons (IDPs) and two [32, 33] amongst refugees.

All studies assessed the impact of WASH interventions on diarrheal disease, with 5 studies occurring or evaluated in relation to general diarrhea [29–33], and one in relation to a suspected—although not laboratory confirmed—*Shigella* outbreak.[34]

The six WASH studies covered multiple interventions. All studies evaluated point of use (POU) treatment, with three [29, 31, 33] focusing on safe water storage (SWS), and two [29, 30] delivering household water treatment (HWT) in the form of disinfection (e.g., flocculent). WASH education [33] and hand washing (including soap provision) (17%) [32] were only evaluated in one study each. Latrine provision and point of source treatment were not evaluated in relation to diarrheal outcomes, although a large-scale study of WASH interventions in a natural disaster (hurricane) in four Latin American countries did report improvements in sanitation in relation to programmatic aims.[31] None of the six studies included in this review explicitly mentioned the Centers for Disease Control and Prevention (CDC) / Pan American Health Organization (PAHO) Safe Water System—a combined WASH package of point of use, safe water storage (SWS), and behavior change interventions—although it is possible that some studies (e.g., Moll et al) may have informally measured aspects of this system [http://www.cdc.gov/safewater/].

### Study designs and quality of research

Four studies [30–32, 34] used an uncontrolled longitudinal study designs, while two [29, 33] were unblinded randomized controlled trials (RCTs). All six studies reported delivery and impact of an intervention, but only one study [32] reported behavior change. It was difficult to assess the reliability of data on uptake and/or compliance from the six studies, and none of the six studies explicitly of thoroughly measured uptake and use of the intervention. One study [32] employing soap distribution did reported marked differences between self-reported use of soap (levels approaching 30%) and observed presence of soap in the house during visits (levels closer to 10%); though not measuring the same indicator, this was the only study to demonstrate the difference between self-report and actual (observed) ability to use the intervention so was considered to provide some evidence in relation to uptake.

Five studies [29–33] conducted a test of significance between WASH interventions and health outcomes. Of these five papers, two [29, 31] were graded to be of higher (15–16 / 22) quality and three [30, 32, 33] were graded to be of more moderate (10–12 / 22) quality. Only one paper [34] that reported WASH interventions and health outcomes without a test of statistical significance and was deemed to be of low quality. In general, the six papers selected for this review did not provide sufficient detail about their design (including statistical assumptions) and methodology; in general those papers that scored highly provided extensive details on all phases of the research. In contrast, moderate quality studies did provide sufficient detail about certain aspects (e.g., population characteristics) but were often lacking in detail about statistical assumptions and discussions of biases and limitations. The one [34] low quality study included in this review did not offer much detail on the design and execution of a campaign of mass container disinfection, but did offer sufficient details in terms of population and intervention (e.g., dates, numbers of containers) to provide some weak indication of this type of option in these settings. The CONSORT and STROBE checklists, with grading scores for each paper, are provided in S3 Appendix and S4 Appendix, respectively.

### Effectiveness of WASH interventions in humanitarian settings

Given that only six studies met the inclusion criteria of this systematic literature review, and the varying study designs and interventions delivered, quantitative aggregate analysis would not be feasible or provide any meaningful conclusions. The results are therefore summarized descriptively, with details of each study provided in Table 2.

The three studies [29, 33, 34] on SWS indicated that these types of interventions may be effective in controlling diarrheal disease; however because these studies are of varying quality and some contain inherent study design issues (e.g., lack of appropriate controls, potential sample size issues), it is impossible to draw meaningful conclusions in relation to the effectiveness of these SWS interventions on health outcomes. Two [29, 30] of six studies utilized water treatment via flocculent disinfectant, or iodinated water filters; these studies demonstrated that water treatment interventions were statistically significant at controlling diarrheal disease. Water treatment and hygiene measures (soap provision) were the most commonly studied and effective interventions in these settings, albeit given the study limitations (e.g., use of self-reported diarrhea, lack of information on uptake) discussed below. Sanitation interventions were not commonly evaluated (likely due to the fact they are less commonly implemented), although one multi-country study [31] included an evaluation of local improvements to sanitation; thus, this systematic literature review cannot comment on their effectiveness.

Doocy *et al*. conducted an unblinded RCT in a Liberian IDP camp wherein all 400 households were provided a SWS intervention in the form of an ‘improved’ water container, while 200 households were provided an additional POU intervention in the form flocculant disinfectant.[29] The authors used self-reported diarrhea, based on weekly surveys over a three-month study period, as their outcome; clinical diagnosis or laboratory confirmation were not employed. This study demonstrated a 90% reduction in diarrheal disease incidence post-intervention amongst intervention households (i.e., those receiving flocculant disinfectant and ‘improved’ water containers) when compared to controls (i.e., those receiving ‘improved’ water containers only). The authors also reported that diarrheal prevalence was 83% lower amongst intervention (i.e., POU flocculant disinfectant) than control (i.e., SWS only) households when compared against baseline. For intervention households, the effects of SWS appeared to amplify the effect of POU flocculant disinfectant to demonstrate 91% lower diarrheal disease prevalence amongst intervention than control households. Substantial differences existed in the sizes and sanitation attributes of each camp. For instance, the populations of Camp I was substantially larger than that of Camp II, potentially promoting the spread of infection; additionally, nearly 30% of Camp I residents reported having no sanitation compared to only 1% of their Camp II counterparts. Authors also cautioned that diarrheal rates normally coincided with the advent of the rainy season so that reported reductions may not be attributable to the flocculant disinfectant alone. While authors reported 1% non-participation in each trial arm and self-reported compliance rates of 86.5%, they did not directly measure uptake or behavior change via observation. The study was considered of high (15 / 22) quality.

Roberts *et al*. conducted an unblinded RCT measuring not only diarrheal disease but geometric mean fecal coliform levels of household water, enabling authors to relate intervention success to both health and water quality outcomes.[33] The intervention in this case was the provision of an ‘improved bucket’ with a 20-litre capacity for water collection and storage, which was provided to 310 intervention households (compared to 850 controls). The authors used self-reported diarrhea, based on weekly surveys over a four-month study period, as their outcome; clinical diagnosis or laboratory confirmation were not employed. However the study provided supportive water quality outcomes in the form of geometric means of fecal coliform levels. The study reported a 69% reduction in geometric mean fecal coliform levels of household water and a 31% reduction in diarrheal disease amongst children < five years old in those households using the bucket; this association was statistically significant. Study authors report a 100% participation rate for households to be interviewed, but did not provide evidence of uptake or behavior change via observation. The study was considered of moderate (12 / 22) quality.

Moll *et al*. conducted a complex evaluation of WASH activities in four Central American countries, attempting to link WASH interventions with childhood diarrhea outcomes in order to monitor if selected communities had met their post-hurricane goals for a given metric (in this case, diarrheal disease reduction in children under three years old).[31] The authors used only self-reported diarrhea, based on pooled analysis of surveys from 800 households, as their outcome. Various interventions were evaluated, including water system upgrades, hygiene and general WASH education, and sanitation (e.g., latrine provision). Many of the interventions reviewed in this study were components of the CDC/PAHO Safe Water System, but study authors did not formally refer to assessing its components [http://www.cdc.gov/safewater/]. Study design and methodology were explicitly detailed, authors reported that diarrheal prevalence decreased from 35% to 26% between the baseline and final surveys; however it was impossible to identify which specific WASH interventions independently impacted diarrheal prevalence given a particular site’s attributes (e.g., urban/rural), Upon univariate analysis, several WASH indicators (proxies for interventions) appeared to be protective against diarrheal disease; however, none of these indicators held up as independently associated with diarrheal disease reduction. This study did not report uptake or behavior change in relation to the various intervention packages rolled out, rendering it impossible to conclude which of these measures was most successful amongst the various intervention packages that were utilized at the four study locations. The study was considered of high (16 / 22) quality.

The uncontrolled longitudinal study by Peterson et al evaluated soap distribution among 356 Mozambican refugee families in Malawi by conducting interviews every two weeks and visiting households (for direct observation) every four weeks over a four-month period.[32] Study authors found that soap provision was associated with 27% reduced diarrheal disease risk when comparing days when soap was observed versus when it was not observed in the participant household. The study also demonstrated a 25% reduction in diarrheal risk compared to controls amongst those households that used soap on the day prior to being interviewed. The authors used only self-reported diarrhea as their outcome. This was the only study to measure and report uptake/behavior change, and did so via direct observation, with 38% of households reporting soap use on interview days and 10% of households demonstrating soap use on observation days. The study was considered of moderate (12 / 22) quality.

Elsanousi *et al*. distributed household iodinated filter to all 647 eligible adult residents of an IDP camp in Sudan, demonstrating a dramatic reduction in new diarrheal cases presenting to a refugee camp clinic when comparing baseline to four months post intervention.[30] However, this uncontrolled longitudinal study used a convenience sample, and compared camp clinic patients to regional hospital admissions. Study authors reported a reduction in diarrheal prevalence from 15% at baseline (four months pre-intervention) to 2.3% four months post-intervention; compared to hospital admissions, this reduction in clinic visits for diarrheal disease was statistically significant. This study was one of only two studies to report laboratory analysis to provide verification on clinical and self-reported diagnoses; however, these results were not analyzed longitudinally and instead provided a cross-sectional analysis of what other pathogens may be circulating in the host populations at baseline. Study authors suggested their design was suboptimal to be able to recommend this intervention, and instead they called for further research (specifically, an RCT). Study authors reported 100% participation rates as all eligible camp residents received the intervention; authors of this systematic literature review interpret this as evidence of 100% distribution, no measurement of uptake or behavior change via observation was provided. Therefore, while this study provides some indication of the effectiveness of household iodinated filters in a humanitarian setting, it does not support any strong conclusions. The study was considered of moderate (10 / 22) quality.

Walden *et al*. evaluated a large-scale mass container disinfection effort, and provided details on the numbers of containers distributed over five days (13,224) as well as diarrheal incidence.[34]

The authors used clinical admissions of watery or bloody diarrhea as their outcome; *Shigella* was suspected but was only confirmed by a handful of laboratory samples (authors do not indicate what proportion of diarrhea was classified as watery versus bloody). However, this study only provided a graph of diarrheal incidence that also indicated when the intervention was conducted; as such, it was not possible to conclude if this mass container disinfection campaign was effective, though at the very least the authors demonstrated that their campaign coincided with a decreased incidence of diarrhea. The study was considered of poor (5 / 22) quality.

## Discussion

This systematic literature review identified only six published studies that evaluated WASH interventions in relation to public health outcomes over the past 33 years.[29–34] All six studies selected evaluated water-related interventions (e.g., SWS, POU interventions such as flocculant disinfectant), while one study measured hygiene as well.[32] POU water quality interventions were most commonly delivered and studied. None of the studies included in this review provided evidence on the impact of sanitation interventions against health outcomes in humanitarian crises.

Among water-related interventions, two high quality studies indicated that POU interventions at the household level are effective at controlling diarrhea, statistically reducing either prevalence or incidence.[29, 31] This is not surprising, given the observed effectiveness of POU interventions in numerous non-humanitarian setting, although the degree of reduction in diarrhea mortality that these interventions can achieve has been questioned because of the lack of effect in blinded studies).[2, 8] SWS measures—from container provision to water treatment—have been increasingly studied and promoted in stable contexts, but the majority of this research has evaluated SWS interventions against water quality outcomes.[35–48] Three studies, two of high [31, 33] and one low [34] quality focused on or included evaluations of household-level SWS.

Only one study attempted to provide evidence on the effects of a hygiene intervention (soap distribution) on diarrheal outcomes. [32] This study found reductions in risk for diarrheal diseases of 25%, or greater when taking in consideration that not all households had been given soap. Evidence from stable, developing contexts indicates that hand washing and soap provision provide extremely effective (and cost-effective) in reducing diarrheal transmission, with behavior (change) identified as an area on which to focus hygiene interventions.[49, 50] The evidence base on hygiene interventions—including alternatives beyond soap provision (e.g., education)—in humanitarian settings could be greatly increased, including ensuring that uptake is recorded.

No studies were identified that evaluated the effects of hygiene interventions on public health outcomes. The range of possible sanitation interventions—i.e., safe excreta removal—has been detailed in more stable settings.[4–6, 51] No studies were identified that evaluated the effects of hygiene interventions on public health outcomes. The range of possible sanitation interventions—i.e., safe excreta removal—has been detailed in more stable settings.[12] For instance, the ability to construct latrines is easier in stable contexts than when doing so in relation to varying water tables resulting from a tsunami. It should be noted that none of the six studies included in this review formally evaluated the CDC/PAHO Safe Water System (http://www.cdc.gov/safewater/), which—based upon the available WASH literature in stable contexts—recommends a combined delivery of safe water storage (SWS), point of use treatment, and behavior change (e.g., hygiene education).

Two [29, 33] of the six selected studies were unblinded RCTs, while the remaining four [30–32, 34] employed uncontrolled longitudinal study designs the latter of which are commonly considered of lower epidemiological quality. However, the appropriateness of RCTs for evaluating WASH interventions is debated; e.g., apart from logistical issues, many WASH professionals consider it unethical to employ what could be considered a ‘lesser’ intervention in a control arm (e.g., no soap, less water) to any individual.[8, 12, 52] A considerable limitation in the two RCT studies was the use of subjective outcomes of self-reported diarrhea (see below).

All of the six studies in this review used self-reported diarrhea as the outcome measure by which to evaluate the success or failure of WASH interventions. Of the six studies included in this review, only one [33] measured water quality standards (fecal coliform) in addition to self-reported diarrhea, and though the study reported a reduction in fecal coliform, a 69% reduction is often not considered sufficient when a 99 to 99.9% should be expected based on WHO standards.[53] Two [30, 34] of the studies included in this systematic literature review provided varying degrees of laboratory confirmation of a diarrheal pathogen, and only two [30, 34] of six studies collected diarrheal data based on physician diagnosis or clinical admissions. It should be noted, however, that the process of clinical examination or diagnosis was not well documented, and the utilization of laboratory confirmation was uneven and not conducted with any statistical or representative basis.

The use of self-reported diarrhea as a health outcome is of particular concern due to inherent biases in the self-reporting process.[8, 52, 54–56] From a perspective of trying to understand the impact of a given intervention, the biases inherent in using self-reported diarrhea have the potential to over-inflate effect sizes—even in the case of RCTs, if they are unblinded.[8] Only one study included in this review collected periodic information on diarrheal illness in intervals less than one week; in this instance, it could be assumed that study authors were able to minimize recall bias as much as reasonably possible [32] This is a major limitation of the research presented in this review, including those studies on hygiene (soap provision) and water treatment, and renders the study difficult to interpret or provide strong recommendations.

Much more preferable to self-reported diarrhea would be laboratory confirmation but only two studies [30, 34] included here reported laboratory results (in negligible samples), and only two studies linked intervention impacts to clinical admissions [30, 34]. While complex emergencies do not often lend themselves to large-scale pathogen discovery and at times diagnostic sensitivity and specificity for some WASH related pathogens (e.g., hepatitis e) may be debatable, laboratory confirmation is extremely important and something the humanitarian WASH community could work towards. In absence of this, better estimates of diarrheal illness than self-reported diarrheal illness, based on higher quality metrics such as physician diagnosis/clinical admissions, are needed if WASH actors can reliably link their efforts to reductions in disease.

There has been much debate on how to categorize the evidence base of WASH interventions, both in conflict and non-conflict settings.[8, 54, 57, 58] Over three decades ago, Blum and Feacham outlined eight issues that must be considered for the evidence generated in relation to WASH interventions to have methodological and statistical basis: “lack of adequate control, the one to one comparison, confounding variables, health indicator recall, health indicator definition, failure to analyze by age, failure to record usage, and the seasonality of impact variables.”[54] By this set of criteria, none of the studies selected in this review would be deemed as high quality, or being able to provide definitive associations between intervention and health outcome. This is because none of the studies included in this review reported anything related to confounding, or seasonality, only one study [32] reported usage/behavior change, and only two studies analyzed by age. This does not mean that according to this classification the studies included in this review do not provide any evidence of impact, but according to these strict epidemiological criteria, none of the studies included in this review appear to have considered the pertinent epidemiological aspects during their design, execution, and analysis.

Tillet *et al*. proposed a framework by which to evaluate waterborne outbreaks (and WASH interventions), albeit in non-humanitarian settings, that take into account epidemiology, microbiology, and water quality data.[58] By this criteria, the majority of studies, including one of the RCTs, included in this review would be classified as being able to provide evidence that the intervention delivered was ‘probably associated’ with the health outcome measured. This is due to the fact that most studies demonstrated an association between intervention and (reduction of) diarrheal disease, but in this case, most did not provide evidence of the pathogen in both the human host (even by self-report) and the water source / WASH intervention under evaluation. The exception to this rule appears to be the RCT by Roberts *et al*., which provided evidence between an ‘improved bucket’ and reductions in not only human diarrhea but also water quality (geometric mean coliform levels of household water) outcomes [33] It should be noted again, however, that this fecal coliform reduction was suboptimal in terms of what would be considered acceptable at the end of an intervention.

All proposed, publish frameworks recommend a combination of considerations on outcome measures, intervention measures, methodological issues (design, control for confounding), assessing other possibly associated factors—e.g., seasonality or in these contexts most often rainy/dry seasons. A similar issue worth reporting, addressed in only one study, was the issue of migration (even if loss to follow-up was reported); only one author provide information on migration, which in that case was inconsistent between intervention and non-intervention participants.[32] The fact that these issues are not included when considering quality of papers (as this review opted to grade papers based on STROBE and CONSORT criteria) could be considered a limitation of this review, as well as those papers included within. Study authors suggest future WASH research in complex emergencies could take such wider issues, including that of uptake, into account when designing future research.

Most WASH epidemiologists concur that the evidence is incomplete without two key features: evidence on uptake/behavior change, and evidence that links both health outcomes and water quality outcomes (discussed further below).[8, 12, 56] With the exception of the study of soap distribution by Peterson *et al*.[32], the majority of the studies retrieved in this review reported distribution of a given intervention (e.g., flocculant disinfectant) and a potential impact on health outcomes (most often, self-reported diarrhea). However, if uptake is suboptimal, the power to detect a statistically significant impact of a given intervention diminishes incrementally. The one study reporting uptake/behavior change reported demonstrated that while nearly 40% of participants reported regular soap usage, only 10% had any soap in their domestic space by direct observation.[32]

The ability for WASH professionals to link the successes, or failures of their interventions to public health outcomes has been long discussed in the literature, both in emergency and non-emergency settings.[2, 6, 8, 12, 52, 54] Typically, the WASH sector has largely been led by water and sanitation engineers rather than medical professionals.[20–25] This may not be without basis, as water quality outcomes are generally considered to be much less subject to bias than common methods of collecting disease data (e.g., self-report).[55, 56, 59] While several important studies have evaluated the success of WASH intervention success in humanitarian contexts against water quality outcomes [19–25, 60–62], this systematic literature review highlights the extremely limited and relatively weak evidence base related to these successes against health outcomes.

Ideally, the evidence base on the impact of WASH interventions would include both water quality (e.g., geometric mean coliform levels) and health (e.g., diarrhea, the pathogen of which would ideally be laboratory confirmed) outcomes.[12] This systematic literature review found only one of six studies that measured both diarrheal and water quality indicators.[33] By all existing criteria on quality of evidence, this lack of consideration to potential pathways of the WASH related disease transmission—including the inability to currently link a given pathogen in the environment to evidence of that pathogen in the human host—makes it impossible to truly implicate or associate an input (e.g., soap distribution) on an impact (e.g., diarrheal disease reduction) of complex, water associated diseases.

This review chose to use only English and French papers, as it was considered this would capture the majority of papers. However, it is possible that this review missed some papers, significantly in Spanish, Portuguese, or Asian languages that may have detailed interventions in these settings; consultation with humanitarian players indicated that expanding to other languages would not have yielded more papers.

## Conclusions

This systematic literature review found a dearth of high quality evidence for the effectiveness of WASH interventions to address public health outcomes in humanitarian crises. While evidence exists on the effectiveness of WASH interventions in relation to water quality or other WASH indicators, there remain significant gaps in knowledge with regards to the impact of WASH in interventions in relation to health outcomes in humanitarian crises. The difficulty of conducting, let alone evaluating, WASH interventions in humanitarian settings is well appreciated, but the limited number of studies and the methodological shortcomings of existing evidence prohibits definitive confirmation on effectiveness in these settings.[12, 18, 63] Future work in this sector must incorporate both public health and measures of use outcomes to provide evidence that interventions are impacting all routes of disease transmission.

This systematic review highlights a number of key recommendations. In terms of study design, it is recommended that studies should: (i) include both public health and water quality outcomes; (ii) evaluate the effects of WASH interventions on non-diarrheal diseases (e.g., trachoma, vector-borne disease); (iii) characterize uptake and/or behavior change, not just distribution, of an intervention (including use of direct observation rather than self-reported where possible); (iv) stronger study designs, statistical reporting, and addressing confounding; and (iv) include data on feasibility, acceptability, cost-effectiveness and sustainability. In terms of WASH interventions recommendations for evaluation include: (i) evaluating water quality interventions beyond POU (even if only verifying that point of source contamination is negligent); and (ii) evaluating alternatives hygiene interventions beyond soap distribution (e.g., WASH education and hygiene promotion). Greater collaboration between WASH professionals and their health and medical counterparts could yield considerable benefits.

## Supporting Information

S1 Appendix Search Strategy (Example: Embase). (DOCX)

S2 Appendix Websites Accessed for Grey Literature Searches. (DOCX)

S3 Appendix CONSORT Checklist, Including Paper Grading. (DOCX)

S4 Appendix STROBE Checklist, Including Paper Grading. (DOCX)

S5 Appendix PRISMA Checklist. (PDF)
