# Supplementary material for: Evidence on the Effectiveness of Water, Sanitation, and Hygiene (WASH) Interventions on Health Outcomes in Humanitarian Crises: A Systematic Review
Source: PLoS One. 2015 Sep 23;10(9):e0124688. doi: 10.1371/journal.pone.0124688 (PMC4580573; doi:10.1371/journal.pone.0124688)
Supplement: S1 Appendix — (DOCX) [file pone.0124688.s001.docx]

**Appendix I: Search Strategy (Example: Embase)**

1. waste water/ or waste water management/ or water pollution indicator/ or water supply/ or water table/ or storm water/ or water stress/ or water table management/ or water contamination/ or water.mp. or water management/ or surface water/ or surface water hydrology/ or water availability/ or water treatment/ or water pollution/ or water structure/ or waste water treatment plant/ or water loading/ or water deprivation/ or drinking water/ or "water and water related phenomena"/ or water quality/ or water/ or ground water/ or water deficit/ or fresh water/ or water analysis/ or water pollutant/ or waste water recycling/ or water sampling/ or water pollution control/

2. water source.mp.

3. body of water.mp.

4. water bod*.mp.

5. drinking water.mp. or drinking water/

6. fresh water.mp. or fresh water/

7. water pollutant/ or water pollution/ or water quality/

8. sanitation/ or sanitation.mp.

9. sewage disposal/ or sewage treatment plant/ or sewage/ or sewage treatment/ or sewage effluent/ or sewage.mp.

10. septic tank.mp. or septic tank/

11. latrine*.mp.

12. toilet*.mp.

13. feces composition/ or feces analysis/ or feces/ or feces.mp. or liquid feces/ or loose feces/

14. faeces.mp.

15. defecation/ or defecat*.mp.

16. hygiene/ or hygien*.mp.

17. "WASH".mp.

18. watsan.mp.

19. drainage.mp.

20. hand washing.mp. or hand washing/

21. handwashing.mp.

22. hand hygiene.mp.

23. soap/ or soap*.mp.

24. detergent/ or detergent*.mp.

25. bore well.mp.

26. borewell.mp.

27. water provision.mp.

28. fecal oral.mp.

29. faecal oral.mp.

30. defecation/ or open defecation.mp.

31. waste treatment.mp. or waste management/

32. 1 or 2 or 3 or 4 or 5 or 6 or 7 or 8 or 9 or 10 or 11 or 12 or 13 or 14 or 15 or 16 or 17 or 18 or 19 or 20 or 21 or 22 or 23 or 24 or 25 or 26 or 27 or 28 or 29 or 30 or 31

33. exp Disasters/

34. exp Relief Work/

35. Rescue Work/

36. Emergencies/

37. Emergency Medicine/

38. Emergency Medical Services/

39. Disaster Medicine/

40. Mass Casualty Incidents/

41. Emergency Responders/

42. Medical Missions, Official/

43. (humanitarian adj2 (crisis or crises or relief or response or agenc$)).tw.

44. humanitarian.tw.

45. (disaster adj3 (relief or plan$)).tw.

46. ((relief or aid) adj2 work$).tw.

47. Refugees/

48. (refugee or evcuee or evacuated).tw.

49. (displace$ adj2 (force$ or population or human or internal$)).tw.

50. Altruism/

51. exp War/

52. war.tw.

53. ((armed or zone) adj2 conflict$).tw.

54. (conflict affected adj3 (population$ or person$ or communit$)).tw.

55. Avalanches/

56. Earthquakes/

57. Floods/

58. Landslides/

59. Tidal Waves/

60. Tsunamis/

61. Cyclonic Storms/

62. (typhoon$ or hurricane$ or cyclone$).tw.

63. (avalanche$ or earthquake$ or flood or floods or flooding or flooded or landslide$ or tsunami$).tw.

64. (disaster adj2 (natural or victim)).tw.

65. Droughts/

66. drought$.tw.

67. Starvation/

68. (starvation or famine$).tw.

69. or/33-68

70. randomized controlled trial/

71. controlled clinical trial/

72. cross-sectional studies/

73. case-control studies/

74. cohort studies/

75. pilot studies/

76. (random$ or controlled).tw.

77. (control adj3 (area or cohort? or compare? or condition or design or group? or intervention? or participant? or study)).ab. not (controlled clinical trial or randomized controlled trial).pt.

78. ((evaluat$ or prospective or retrospective) adj1 study).tw.

79. ("quasi-experiment$" or quasiexperiment$ or "quasi random$" or quasirandom$ or "quasi control$" or quasicontrol$ or ((quasi$ or experimental) adj3 (method$ or study or trial or design$))).tw.

80. ("time series" adj2 interrupt$).tw.

81. (intervention$ or impact or effectiveness or efficacy or service$ or outcome$ or output or treatment$ or management or program$ or project$).tw.

82. economics/

83. cost-benefit analysis/

84. cost control/

85. Cost savings/

86. cost of illness/

87. cost $utility.tw.

88. (Cost$ adj2 effective$).tw.

89. cost-effective$.tw.

90. (cost adj3 utility).tw.

91. cost-utilit$.tw.

92. or/70-91

93. developing countries/

94. exp asia/

95. exp africa/

96. exp pacific islands/

97. caribbean region/ or central america/ or latin america/ or south america/

98. exp eastern europe/

99. exp china/

100. balkan peninsula/ or europe, eastern/ or transcaucasia/

101. americas/ or caribbean region/ or central america/ or "gulf of mexico"/ or latin america/ or south america/

102. antarctic regions/ or atlantic islands/ or indian ocean islands/ or macau/ or pacific islands/ or philippines/ or prince edward island/ or svalbard/ or west indies/

103. or/93-102

104. Japan/

105. 103 not 104

106. 69 and 92 and 105

107. limit 106 to yr="1980 -Current"

108. 32 and 107
