# Supplementary material for: Evidence on the Effectiveness of Water, Sanitation, and Hygiene (WASH) Interventions on Health Outcomes in Humanitarian Crises: A Systematic Review
Source: PLoS One. 2015 Sep 23;10(9):e0124688. doi: 10.1371/journal.pone.0124688 (PMC4580573; doi:10.1371/journal.pone.0124688)

**Appendix II: Websites Accessed for Grey Literature Searches**

- Google
- Research for Development (R4D)
- ReliefWeb
- Desastres
- Eldis
- Active Learning Network for Accountability and Performance (ALNAP)
- Centre for Research on Epidemiology of Disasters (CRED)
- MSF Field Research
- World Health Organization (WHO)
- UN Children’s Fund (UNICEF)
- International Committee of the Red Cross (ICRC)
- MSF Field Research
- UN High Commission for Refugees (UNHCR)
- UN-Habitat
- WaterAid
- SHARE
- UN-Water
- Secretariat of the United Nations Convention to Combat Desertification (UNCCD)
- UN Development Programme (UNDP)
- UN Environmental Programme (UNEP)
- UN International Strategy for Disaster Reduction (UNISDR)
- International Water Resources Association (IWRA)
- Global Water Partnership
- International Water Association (IWA)
- Public Services International (PSI)
- Gender and Water Alliance (GWA)
- Women for Water Partnership
- Conservation International
- Water.org
- UN International Groundwater Resources Assessment Centre (IGRAC)


Supplement: S2 Appendix — (DOCX) [file pone.0124688.s002.docx]
