# Supplementary material for: Evidence on the Effectiveness of Water, Sanitation, and Hygiene (WASH) Interventions on Health Outcomes in Humanitarian Crises: A Systematic Review
Source: PLoS One. 2015 Sep 23;10(9):e0124688. doi: 10.1371/journal.pone.0124688 (PMC4580573; doi:10.1371/journal.pone.0124688)
Supplement: S4 Appendix — (DOCX) [file pone.0124688.s004.docx]

**Appendix IV: STROBE Checklist, Including Paper Grading**

| **STROBE** | **Item No** | **Recommendation** | **Elanousi et al.** | **Moll et al.** | **Peterson et al.** | **Walden et al.** |
| --- | --- | --- | --- | --- | --- | --- |
|  |  |  | **(X)** | **(X)** | **(X)** | **(X)** |
| **Title and abstract** | | | | | |  |
|  | 1 | (a) Indicate the study’s design with a commonly used term in the title or the abstract |  |  |  |  |
|  |  | (b) Provide in the abstract an informative and balanced summary of what was done and what was found | X | X | X |  |
| **Introduction** | | | | | | |
| Background / rationale | 2 | Explain the scientific background and rationale for the investigation being reported | X | X | X | X |
| Objectives | 3 | State specific objectives, including any prespecified hypotheses | X | X |  |  |
| **Methods** |  |  |  |  |  |  |
| Study design | 4 | Present key elements of study design early in the paper | X |  | X |  |
| Setting | 5 | Describe the setting, locations, and relevant dates, including periods of recruitment, exposure, follow-up, and data collection | X | X |  | X |
| Participants | 6 | a) Cohort study: Give the eligibility criteria, and the sources and methods of selection of participants. Describe methods of follow-up. Case-control study: Give the eligibility criteria, and the sources and methods of case ascertainment and control selection. Give the rationale for the choice of cases and controls. Cross sectional study: Give the eligibility criteria, and the sources and methods of selection of participants |  | X |  |  |
|  |  | b) Cohort study: For matched studies, give matching criteria and number of exposed and unexposed Case-control study: For matched studies, give matching criteria and the number of controls per case |  |  |  |  |
| Background / rationale | 7 | Clearly define all outcomes, exposures, predictors, potential confounders, and effect modifiers. Give diagnostic criteria, if applicable |  |  | X |  |
| Data sources/ measurement | 8 | For each variable of interest, give sources of data and details of methods of assessment (measurement). Describe comparability of assessment methods if there is more than one group |  | X |  |  |
| Bias | 9 | Describe any efforts to address potential sources of bias |  |  |  |  |
| Study size | 10 | Explain how the study size was arrived at |  | X |  | X |
| Quantitative variables | 11 | Explain how quantitative variables were handled in the analyses. If applicable, describe which groupings were chosen and why | X |  | X |  |
| Statistical methods | 12 | a) Describe all statistical methods, including those used to control for confounding |  | X | X |  |
|  |  | (b) Describe any methods used to examine subgroups and interactions |  |  |  |  |
|  |  | (c) Explain how missing data were addressed |  | X |  |  |
|  |  | (d) Cohort study:If applicable, explain how loss to follow-up was addressed |  |  |  |  |
|  |  | Case-control study:If applicable, explain how matching of cases and controls was addressed |  |  |  |  |
|  |  | Cross sectional study:If applicable, describe analytical methods taking account of sampling strategy |  |  |  |  |
|  |  | (e) Describe any sensitivity analyses |  |  |  |  |
| **Results** |  |  |  |  |  |  |
| Participants | 13 | a) Report numbers of individuals at each stage of study: eg numbers potentially eligible, examined for eligibility, confirmed eligible, included in the study, completing follow-up, and analysed | X |  |  |  |
|  |  | (b) Give reasons for non-participation at each stage |  |  |  |  |
|  |  | (c) Consider use of a flow diagram |  |  |  |  |
| Descriptive data | 14 | a) Give characteristics of study participants (eg demographic, clinical, social) and information on exposures and potential confounders |  | X | X |  |
|  |  | b) Indicate number of participants with missing data for each variable of interest |  |  |  |  |
|  |  | (c) Cohort study:Summarise follow-up time (eg average and total amount) |  |  |  |  |
| Outcome data | 15 | Cohort study:Report numbers of outcome events or summary measures over time | X |  | X |  |
|  |  | Case-control study:Report numbers in each exposure category, or summary measures of exposure |  |  |  |  |
|  |  | Cross sectional study:Report numbers of outcome events or summary measures |  | X |  |  |
| Main results | 16 | (a) Report the numbers of individuals at each stage of the study:eg numbers potentially eligible, examined for eligibility, confirmed eligible, included in the study, completing follow-up, and analysed |  | X | X |  |
|  |  | (b) Give reasons for non-participation at each stage |  |  |  |  |
|  |  | (c) Consider use of a flow diagram |  |  |  |  |
| Other analyses | 17 | Report other analyses done:eg analyses of subgroups and interactions, and sensitivity analyses |  | X |  |  |
| **Discussion** | | | | | | |
| Key results | 18 | Summarise key results with reference to study objectives | X | X | X |  |
| Limitations | 19 | Discuss limitations of the study, taking into account sources of potential bias or imprecision. Discuss both direction and magnitude of any potential bias |  | X | X | X |
| Interpretation | 20 | Give a cautious overall interpretation of results considering objectives, limitations, multiplicity of analyses, results from similar studies, and other relevant evidence |  | X | X |  |
| Generalisability | 21 | Discuss the generalisability (external validity) of the study results |  |  |  |  |
| **Other information** | | | | | | |
| Funding | 22 | Give the source of funding and the role of the funders for the present study and, if applicable, for the original study on which the present article is based | X |  |  | X |
|  |  | **TOTAL SCORES** | **10** | **16** | **12** | **5** |
|  |  |  | *Elanousi et al.* | *Moll et al.* | *Peterson et al.* | *Walden et al.* |
|  |  |  | *(X)* | *(X)* | *(X)* | *(X)* |
